# Wages versus Comfort

**Published:** 1892-05-07

**Authors:** 


### Wages versus Comfort.

### A SCOTTISH SKETCH.

It is a common remark with those who stand forth most prominently in public as the friends of the working class that "capital" compels "labour" to accept a bare subsistence as wages. It may be that in the estimate of "labour" this is true enough, for "bare subsistence" like "competence" is a phrase of variable meaning, and usually stands for the exact amount of one's income. But when the gaunt spectre of half-starved labour is thrust upon one's imagination, it is worth while to compare the wage of some working-men with that of others who—because of the popular theory that broad-cloth, however shabby, implies idleness in the wearer—never get credit for doing work at all.

If an explosion occurs in a coal-pit, and kills some of the miners, a subscription is immediately started for the widows and orphans, who are invariably left utterly destitute. No one ever heard of a subscription for the widows and orphans of—say—defunct clerks and counter-jumpers. For the latter are supposed to be "passing rich on forty pounds a year," while the poor miners have been struggling along on a "bare subsistence" of about a hundred and fifty. It is perfectly true, nevertheless, that the widows and orphans are destitute, for the traditions of the mining craft forbid anything approaching the sordid virtues of economy or providence. If a man earns more in a week than he can conveniently spend in meat and drink—especially drink—he works the less next week to make up for it. But this does not happen often, for if there be a creature who can consume more intoxicating liquor than a collier, it is a collier's wife. This capacity is almost the only one she possesses. Of those domestic accomplishments which are supposed to be innate in every woman's breast she shows hardly a trace; of the domestic affections she possesses rather less than the average cat. A visit to the home of a Lanarkshire collier would justify the "friend of humanity" in his assumption that its occupant earned no more than a bare subsistence, and it is difficult for anyone who does not know what the miner is to believe that even a moderate wage is spent in such a house.

Usually it consists of but a single room, and that so squalid that one would suppose soap and a scrubbing brush to be luxuries too dear for the occupant's purse. For furniture there is the inevitable box-bed—somewhat dilapidated, perhaps, because its frame is so handy for converting into firewood—covered with foul blankets and a yet more filthy quilt. The master of the house would scorn to wash himself before lying down, and one miner who, by some accident, had wandered to an English colliery, relates with horror that he lodged with a woman who insisted on his undergoing a "tubbing" before he went to bed. Besides this, there is a ricketty table and perhaps one or two shaky chairs. It is likely enough, however, that the latter are wanting, and that a block of coal supplies all that is needed in the way of seats. For summer there is the doorstep, for winter the lump of coal in the ingle-neuk. What more do these children of nature want?

In spite, however, of this lack of luxury in her surroundings, the collier's wife enjoys an existence infinitely lazier than that of the average duchess. She toils not, neither does she spin, nor mend her clothes, nor clean her house, nor see to the ways of her household. The frying-pan is her only domestic utensil, and it she uses in rough and unready fashion; her children thrive or die on a regimen of blows, mitigated by bread and jam. She has her amusements—intoxication and an occasional lottery, when the "row" or village is incited by the pedlar of bright-hued skirts and tawdry earrings of dubious gold. Then with some half-dozen of her neighbours she will unite in the purchase of the article desired, and draw lots who shall get it. It is a matter of etiquette that the fortunate winner shall take part in the next "jine," as it is called, or refrain from claiming the prize; but, unfortunately, this rule is not always kept, and she who has gained the trinket this week will refuse her contribution to the next week's deal. Thence come words—nay, the collier woman does not confine herself to words, but goes on to blows and the tearing of hair, till a reconciliation is brought about by neutral parties, and cemented by ardent spirits.

"As the husband is the wife is," but is it strange that the husband of such a wife should be as bad as she? There is little to choose between them, though the husband claims the marital right of cursing his wife, beating her, and occasionally, when full of wrath and potato spirit, kicking her to death. Your collier, or iron worker, likes potato spirit, because it affords a swifter and more maddening intoxication than the more legitimate liquor; for it is not the pleasure of the palate that he seeks, but delirium of the brain. He cannot conceive an existence that is not flooded with strong waters, and his children regard drunkenness as an integral part of life. Witness the following sentence, written by a girl of ten in a school exercise, which purported to give some account of a newly-established iron-work: "The men require to take a lot of drink with them because it is so hot."

One might almost say that it is only on the side of their vices that these people touch humanity. On many points one would be glad to plead for them the irresponsibility of sheer animalism. The relations of the sexes might satisfy Ouida, but are a standing distress to the clergy of all denominations who strive to improve the miners' morals. Things are indeed better than they were a generation since, when absolute promiscuity obtained in the remoter villages, and the children born during the preceding year were allotted every first of January to those whom they were in the future to regard as fathers. Happy the man who could secure a lusty boy as his share! For boys are a fortune to the miner, as he implies when he nicknames one a "hutch o' roond." A girl is only a "hutch o' dross." A boy of fourteen can earn ten shillings a week; nay, by a little diplomacy, he may be made to gain as much in a day. The output of each man is restricted; but it is not forbidden to a father to help his son to do a man's work and earn a man's wage. Thus to these squalid houses that look so poverty-stricken there often comes as much as four, five, or six pounds a week. There is much truth in the remark recently made by a dissenting minister whose lot has fallen among colliers, "We have no poverty here; but we have abundance of misery."

Truly theirs is disheartening work, the priests, ministers, and schoolmasters who labour among these people. They persuade a man to take a temperance pledge, and he gets drunk on the next pay-night. The priest uses every threat his creed permits to drive into lawful bonds some couple who have been living together comfortably enough without the Church's benediction, and finds that once they are safely married fights and quarrels begin. Even when some respect for the legitimate tie exists it does not imply any very high moral tone. Recently a woman, whose husband was buried on Friday, married another man on Saturday, and left this second spouse before a week was past—lightly come and lightly go! The teacher who has driven a child up to the fifth standard which gives freedom, finds that in a year or two his pupil cannot even read, and has to learn his letters over again. That the pupil is occasionally willing to go through the drudgery of learning again, even at the end of a long and dreary day's work, implies, however, something of a better spirit. Occasionally some indication tells that education has not been wholly given in vain. One young collier has even produced a poem. The first stanza runs thus:—

"Oor hoose is a reeky hoose,
The reekiest in the Raw;
Jock pu'd oot a half-brick,
And let the reek awa'."

*Le rime n'est pas riche*, but it shows an embryonic notion of rhyme and rhythm. There is, besides, the dawning consciousness of the unpleasantness of "reek" in a dwelling. Jock has begun to perceive and think; he is not wholly past redemption.

